# Congenital occipital myelocele

**DOI:** 10.1590/S1679-45082018AI4123

**Published:** 2018-04-19

**Authors:** José Ricardo Dias Bertagnon, Marcos Augusto Cruz Rocha, Marina Affonso dos Santos Fonseca Ribeiro, Manuella Pedroza Limongi

**Affiliations:** 1 Universidade de Santo Amaro, São Paulo, SP Brazil.

## INTRODUCTION

Congenital encephalocele is a neural tube closure defect that presents herniation of cranial contents because of a cranial congenital malformation, the occipital encephalocele is the most common form of this defect.^(^ ^1^ ^)^ Encephalocele often occur because of occurrence of occipital bone defect within the fourth week of embryogenesis and it can be extended for foramen magnum and affect the posterior arch of the atlas.^(^ ^2^ ^)^ Hernia sac content varies and worst prognosis is large brain content inside the sac.^(^ ^3^ ^,^ ^4^ ^)^ Etiology of this malformation include genetic and environmental factors, such as folate deficiency that is prevented with the use of folic acid supplements during preconception period until 12 weeks of gestation, and also for poor prenatal care.^(^ ^5^ ^,^ ^6^ ^)^ Although this malformation varies in several demographic regions, neural tube closure defects incidence is approximately 1 in 1,000 live births.^(^ ^7^ ^,^ ^8^ ^)^

Both adequate prenatal care and imaging tests are importance to identify variations of this affection. For this reason, clinical case reports and their on encephalocele images are important.

## DESCRIPTION OF CLINICAL CASE

A 19-years-old healthy primigravida mother with no remarkable clinical history, who reported no use of folic acid or iron supplementation during the preconception and prenatal period.

During prenatal period, ultrasonography tests according to second and third trimester of gestation showed a single and live fetus with cystic formation in posterior cervical region that could indicate a cystic hygroma and occipital encephalocele, in addition to severe bilateral ventriculomegaly.

**Figure 1:**
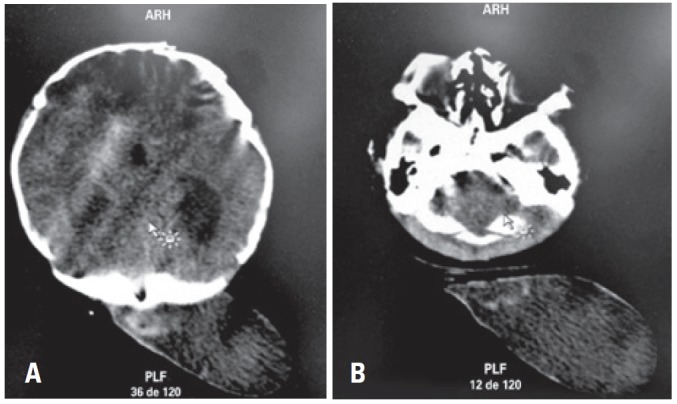
Cranial computed tomography with occipital subcutaneous cystic formation to the left with licorice attenuation of content associated with occipital bone micropuncture in the midline and cortex isodense mass, therefore, suggesting encephalomeningocele

Because of the features of the case, we decided to undertake a cesarean section in week 39 of gestation. This was a female newborn weighting 3,100g, measuring 45cm, with head circumference of 33cm and Apgar score of 9.9. The patient evolved clinically stable without intercurrences. However, an encephalocele was observed (Figure 1).

**Figure 2:**
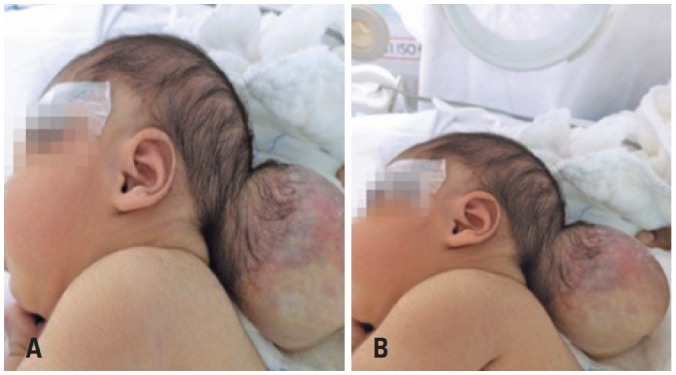
Newborn with hernia sac in occipital region

**Figure 3:**
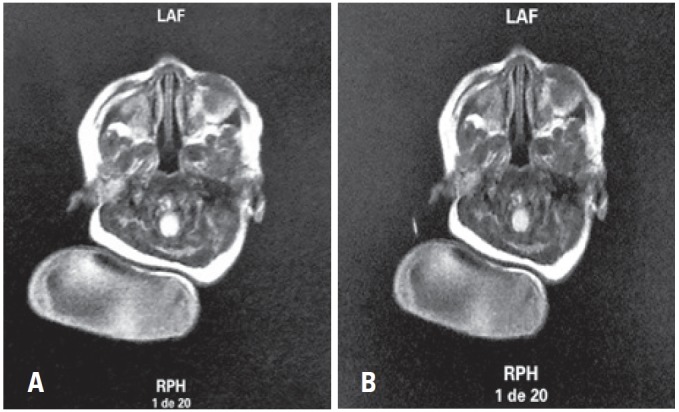
Cranial magnetic resonance imaging showing median suboccipital bone defect with extrusion of superior aspect and cerebellar vermis, cerebrospinal fluid and meninges (encephalomeningocele)

A computed tomography (Figure 2) and a cranial magnetic resonance imaging (Figure 3) were requested after the child birth. Tests results showed occipital encephalomeningocele with median suboccipital bone defect with extrusion of content similar to cerebrospinal fluid and meninges.

## DISCUSSION

Encephalomeningocele is a congenital malformation caused by neural tube closure defect. This disease has a poor prognosis because it can cause infection in central nervous system, in addition to advanced stages motor and sensory deficiencies.^(^ ^9^ ^,^ ^10^ ^)^

Cranial computed tomography and magnetic resonance imaging are tests used to diagnose this type of disease, define hernia sac content, and to evaluate the best surgical approach.^(^ ^9^ ^)^

In our case, the mother did not receive folic acid supplementation during preconceptional period up to 12 weeks of gestation, which is a risk factor for evolving of this disease^(^ ^5^ ^)^ in newborns.

Of note, to perform imaging tests adequate to gestational age is paramount to diagnose as early as possible affections such as the encephalomeningocele.

During prenatal tests a clinical feature compatible with encephalocele was identified, but only after the birth more specific tests were done such as cranial computed tomography and magnetic resonance imaging. Results showed a clinical picture different from encephalocele, the encephalomeningocele, being this latter more severe and with worse prognosis because it evolves a larger parcel of cerebral content, such as brain and meninges.^(^ ^9^ ^)^

## References

[1] Northrop H, Volcik KA (2000). Spina bifida and other neural tube defects. Curr Probl Pediatr..

[2] Walia B, Bhargava P, Sandhu K (2005). Giant occipital Encephalocele. Med J Armed Forces India..

[3] Aguiar MJ, Campos AS, Aguiar RA, Lana AM, Magalhães RL, Babeto LT (2003). Defeitos de fechamento do tubo neural e fatores associados em recémnascidos vivos e natimortos. J Pediatr..

[4] Lorber J, Schofield JK (1979). The prognosis of occipital encephalocele. Z Kinderchir Grenzgeb..

[5] Copp AJ, Greene ND (2010). Genetics and development of neural tube defects. J Pathol..

[6] Prevention of neural tube defects: results of the Medical Research Council Vitamin Study (1991). MRC Vitamin Study Research Group. Lancet..

[7] Botto LD, Moore CA, Khoury MJ, Erickson JD (1999). Neural-tube defects. N Engl J Med..

[8] Melvin EC, George TM, Worley G, Franklin A, Mackey J, Viles K, Shah N, Drake CR, Enterline DS, McLone D, Nye J, Oakes WJ, McLaughlin C, Walker ML, Peterson P, Brei T, Buran C, Aben J, Ohm B, Bermans I, Qumsiyeh M, Vance J, Pericak-Vance MA, Speer MC (2000). Genetic studies in neural tube defects. NTD Collaborative Group. Pediatr Neurosurg..

[9] Kinsman SL, Johnston MV, Kliegman RM, Behrman RE, Jenson HB, Stanton BF (2007). Congenital anomalies of the central nervous system. Nelson Textbook of Pediatrics.

[10] Pinto AP, Gomes C, Faria CC, Faria JM, Saldanha J (2016). Encephalomeningocele: Inside the Picture. Acta Med Port..

